# Protocol-Based and Hybrid Access Control for the IoT: Approaches and Research Opportunities

**DOI:** 10.3390/s21206832

**Published:** 2021-10-14

**Authors:** Shantanu Pal, Zahra Jadidi

**Affiliations:** 1 School of Computer Science, Faculty of Science, Queensland University of Technology, Brisbane, QLD 4000, Australia; 2 Cyber Security Cooperative Research Centre, Queensland University of Technology, Brisbane, QLD 4000, Australia; zahra.jadidi@qut.edu.au

**Keywords:** Internet of Things, access control, policy management, security, architecture

## Abstract

Internet of Things (IoT) applications and services are becoming more prevalent in our everyday life. However, such an interconnected network of intelligent physical entities needs appropriate security to sensitive information. That said, the need for proper authentication and authorization is paramount. Access control is in the front line of such mechanisms. Access control determines the use of resources only to the specified and authorized users based on appropriate policy enforcement. IoT demands more sophisticated access control in terms of its usability and efficiency in protecting sensitive information. This conveys the need for access control to serve system-specific requirements and be flexibly combined with other access control approaches. In this paper, we discuss the potential for employing protocol-based and hybrid access control for IoT systems and examine how that can overcome the limitations of traditional access control mechanisms. We also focus on the key benefits and constraints of this integration. Our work further enhances the need to build hierarchical access control for large-scale IoT systems (e.g., Industrial IoT (IIoT) settings) with protocol-based and hybrid access control approaches. We, moreover, list the associated open issues to make such approaches efficient for access control in large-scale IoT systems.

## 1. Introduction

With the rapid improvements in intelligent sensors, wireless sensor networks, and smart mobile devices, there has been a huge growth in the number of devices per user in recent years. These devices can be interconnected to sense, monitor, and play in autonomous decision making as well as exchange information in both physical and digital systems. Such a system that combines interconnected devices, objects, and humans and that is able to collect and transfer data over the network is commonly recognized as Internet of Things (IoT). IoT can be used to manage such a large number of interconnected devices providing specific services [1,2].

Although IoT has the potential to make the internet infrastructure more scalable and flexible, using its dynamic characteristics and mobility present in interactions, the resource-constrained nature of IoT devices introduces new security vulnerabilities [3]. Some of these vulnerabilities are related to authentication (i.e., verifying the identity of an entity) and authorization (i.e., if the entity has the permission to access a specific resource), including insecure access to the web, back-end APIs, cloud, and mobile interfaces [4].

**Figure 1:**
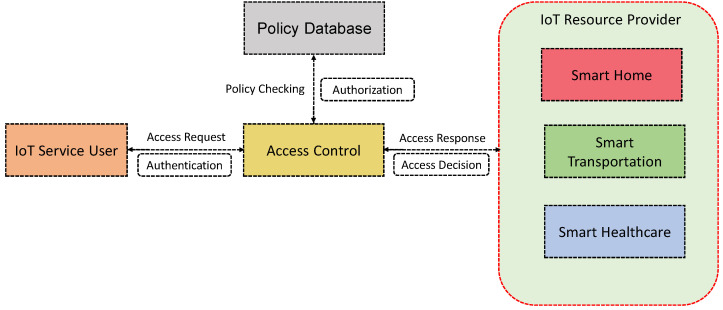
A typical outline of IoT access control scenario.

One of the important security concerns in IoT is access control [5]. Access control in the IoT plays an important role to ensure the system’s efficiency and performance. It is a security mechanism that guarantees the availability, integrity, and confidentiality of resources. It determines how and in what way a legitimate entity can access a resource. In other words, access control determines the permissions to a certain access to a certain entity based on the policies and incorporated conditions set for this particular access. That said, placing appropriate authentication and authorization techniques at the front line is significant [6,7]. In Figure 1, we illustrate a simple outline of an IoT access control scenario. In an interconnected IoT system, access control should be enforced to prevent unauthorized access from vulnerable devices and machines as well as human users. An appropriate access control mechanism can be utilized to monitor the access activities to the devices and ensure that unauthorized users cannot exploit devices and apps and gain access to users and devices’ data [2,8,9,10]. IoT devices transmit and share data to achieve a specific goal mostly in a resource constrained environment. This possesses some restrictions, where the traditional access control methods are not fully applicable to the IoT environment, given the nature, dynamics, and specific characteristics present in the IoT [11,12]. In other words, IoT makes it challenging to employ well-established security mechanisms, due to its resource-limited nature of battery power, memory space, and processing speed. Further, the other characteristics of an IoT system (e.g., high mobility, dynamic network topology, and heterogeneity in devices) show significant demand, where the need for access control can be placed in a combination of one or more network protocols or even access control mechanisms. That said, an IoT system needs to be dedicated with context-specific access control mechanisms to manage the users, applications, services, and their complex associations [13,14].

### 1.1. Motivation and Problem Statement

Now consider a situation of a roadside emergency service during an accident. Intelligence signaling systems may capture the incident and call emergency response services automatically. At the accident place, police officers can communicate with the other emergency services (e.g., fire services) and inform hospital authorities in advance of the present condition of the driver or passengers. However, managing this information over the jurisdictions possesses various interdisciplinary challenges associated with service providers and the combination of information coming from various smart sensors. They may need specific access control mechanisms to seamlessly combine multiple protocols and access control mechanisms to deliver a better service [15,16].

There are several commonly known access control mechanisms that are widely employed for IoT access control based on the system’s requirements and the designer’s choice. For example, role-based access control (RBAC) is used for IoT access control. While the use of RBAC is promising when requiring stronger security (by enforcing effective policy management), its use in the context of the IoT is limited (i.e., within a closed system). The policy management and their informants in RABC are highly centralized and, therefore, it imposes several limitations when designing access control for a highly dynamic and scalable system, such as the IoT. In RBAC, roles are mapped to permissions, and permissions are then assigned to certain users. It typically demands precise user assignment to specific roles and holds only pre-defined, including static, policies. In an IoT context, this is difficult given the high dynamics (for both users and devices) present in the system [17].

Another commonly used access control mechanism for IoT systems is attribute-based access control (ABAC). It supports much flexibility in access control, as the permission decisions are performed based on attributes [18]. Unlike RBAC, ABAC reinforces the scale issue in an IoT system to a higher extent. Attributes can be referred to as the certain properties of an entity. For instance, the attribute of an entity (e.g., a person, Alice) can be a set of properties that may uniquely define the entity within a given context (e.g., Alice’s date of birth, driving license number, and phone number). The use of attributes supports fine-grained access control to the IoT. However, it raises important questions of policy evaluations and associated high costs of applications for policy evaluation [19].

The use of capability-based access control (CapBAC) is gaining more focus in the IoT. In CapBAC, the access control rights are transferred in the forms of capabilities (also known as access tokens). One of the fundamental advantages of CapBAC is that it considers the resource-constrained characteristics of IoT devices. That is, in CapBAC, the access control policy and rules are embedded inside a capability that can be evaluated locally at the edge IoT devices. In other words, the edge devices can perform local authorization checking and determine access control decisions in real time without the need for a centralized authorization system. However, in CaBAC, establishing trust among the entities is a significant issue [14].

When the ABAC, RBAC, and CapBAC try to address the authorization issues in general, several approaches are discussed to inspect the authentication and secure communication issues to address dedicated security issues in the IoT systems at the protocol level [20,21], for instance, the use of reliable, lightweight communication protocols for the IoT, e.g., datagram transport layer security (DTLS) with the existing internet standards. In many cases, the constrained application protocol (CoAP)–based framework is also applied to accomplish a fine-grained access control focused on low overhead for resource-constrained IoT devices. As noted above, the resource-constrained characteristics of these devices make it difficult, where traditional heavy-weight security solutions are not feasible to employ directly for them. It emphasizes the critical issue of securing communication and authentication and their secure interactions by considering lightweight security explications and essential management techniques by dedicated protocol-based access control solutions. The *Protocol-Based Access Control (ProBAC)* can be referred to as the specific selection of protocols used for IoT access control to serve particular access control needs.

To overcome the limitations of individual access control mechanisms, a trend tries to combine two or more access control mechanisms and introduce a *Hybrid Access Control (HyBAC)* approach. That is, an HyBAC combines the properties of two or more access control solutions and takes advantage of each of them. We note that a HyBAC model serves specific purposes of access control within a given context. For instance, the combination of RBAC and ABAC mechanisms together with IoT access control. A few other proposals combine ABAC and a distributed trust management framework. However, it must be noted that these proposals are not a complete solution for approaching the IoT access control problem by obtaining the advantages of an individual access control mechanism. The choice of a particular protocol or hybrid access control model is context dependent and depends upon the specific requirements of the systems.

We argue that such a consideration of ProBAC and HyBAC would be beneficial when considering large-scale IoT systems, for example, the industrial Internet of Things (IIoT) applications [22]. It is a significant challenge for grating access to critical assets more intelligently and faster in such IIoT systems. In general, IIoT helps increase quality in performance and safety to a greater extent. However, due to the different nature of the IIoT environment, they need different strategies for access control. When combining machine-to-machine (M2M) communication for reliable and efficient industrial data analytics and accelerating digital data transformation, the levels of efficiency, productivity, and performance in access control are pre-eminent. Therefore, it directs the need for hierarchical access control that must be placed at each layer of an IIoT architecture. This paper also aims to examine the importance and design consideration of such hierarchical access control for large-scale IoT systems.

### 1.2. Contributions

This paper presents a review of the existing models and methods for ProBAC and HyBAC for IoT systems. Our study aims to show the importance of considering protocols and their lightweight implementations and combining one or two access control methods to provide more flexibility to access control in IoT. This is specifically critical, given the resource-limited nature of the IoT devices. Several proposals review access control issues in the IoT within the scope of traditional access control mechanisms. In Table 1, we illustrate the comparison of our work with the existing reviews in IoT access control. For instance, Fotiou et al. [23] discuss an overview of access control in the IoT context. In this proposal, access control architecture is discussed from a centralized perspective. Therefore, the discussion of this proposal is limited only to a particular access control architecture (i.e., centralized) that uses a lightweight authentication scheme, using symmetric key cryptography. Furthermore, the discussion is limited to the generally employed access control methods, e.g., RBAC, ABAC, and CapBAC.

In [24], Elsayed et al. provide a comprehensive survey of different access control models used for pervasive environments. This survey provides access control aspects from the security requirements point of view. Three commonly known security requirements, e.g., confidentiality, integrity, and availability, are considered. Moreover, it classifies various access control models into four distinct classes. They are context-aware, attribute-based, user behavior-based, and relation-based. Then, a comparison is made among these four classes based on eight particular issues present in an IoT system. They are dynamic, flexible, personalize, adaptive, extensive, context-aware, trust, private, smart, and relation. However, the comparisons are made on a very high level, and how these comparisons could lead toward a foundation of a secure access control model for the IoT is not demonstrated. Furthermore, it focuses more on general pervasive environments, and no focus is given to significant IoT-related protocol and hybrid access control issues.

In [25], Ranjan and Somani provide a survey in access control and authentication in an IoT environment. Unlike [24], this survey provides a discussion on different access control issues based on the security concerns present in three distinct layers of an IoT architecture, i.e., perception layer, transportation layer, and application layer. The major contribution of the literature is a systematic comparison between various proposals discussing authentication and authorization based on the technique used, whether they are implemented in real time and if they provide security analysis. However, the survey mainly discusses access control from an architectural layer perspective.

In [26], Zhang and Wu provide a survey on access control in the IoT. The major focus of the survey is to build an access control model that is based on trust computing, where trust establishment happens between the devices. However, the trust establishment is performed in a highly centralized system. Alramadhan and Sha [27] present an overview of access control mechanisms for the IoT. The discussion is limited to commonly used access control mechanisms, e.g., RBAC, ABAC, and CapBAC. It leaves out many important aspects of access control mechanisms, e.g., decentralization, scalability, and availability. Rvaidas et al. [28] present a survey for access control in the context of an IoT system. It focuses on the requirements of authorization frameworks for IoT systems. Three distinct components assess these authorization frameworks. They are policy specification, policy management, and policy evaluation and enforcement. It discusses to what extent the existing authorization frameworks meet the IoT access control requirements. The survey presents a detailed discussion on different IoT architectures and enabling technologies in each layer. However, the survey is more focused on policy enforcement and their evaluation of certain access control mechanisms for the IoT within the scope of a highly centralized system. Bertin et al. [29] discuss existing access control approaches and present a set of open research questions for the IoT. The proposal is focused on different access control models and their suitability in IoT systems. However, the survey mainly addresses some issues (e.g., lightweight M2M protocol and personal data handling) related to IoT security. Once again, these proposals do not provide a discussion of protocol and hybrid access control models used for the IoT.

Qiu et al. [30] present a survey on access control in the edge IoT. The major focus is to survey the existing access control models used for IoT search technology. IoT search technology is used for gathering information quickly and accurately based on the real-time search needs of the users. However, the discussion is restricted to access control policy composition (including policy standardization and conflict resolution) and access control policy authoring (including permission assignment and policy matching). It does not consider the other access control requirements (e.g., mobility, integration, and data trust) for the IoT. In [31], Ouaddah et al. present a survey on access control in the IoT focused on access control models, protocols, and frameworks. First, a taxonomy of access control is presented. Then, several existing access control models are analyzed, based on the 11 characteristics related to an IoT system: scalability, usability, flexibility, interoperability, context-awareness, distribution, real-time, heterogeneity light-weight, user-driven, and granularity. In [32], Ouaddah et al. extend the work presented in [31] and survey IoT access control in a more structured way. In the survey, an in-depth review of different access control solutions in the IoT is discussed based on their objectives, models, architectures, and mechanisms. Alnefaie et al. [33] present a survey on IoT access control focused on security-specific requirements. Again, all of the discussions are limited to the traditional approach of access control approaches to IoT systems without focusing on protocols and hybrid access control approaches.

Unlike the existing surveys, in this paper, we review the existing access control mechanisms for the IoT based on protocol and hybrid approaches. The current surveys in IoT access control mainly focused on the traditional access control methods, e.g., RBAC, ABAC, and CapBAC. While these conventional models provide some robust features, such as the strong security in RBAC and dynamic behavior of ABAC [15], they have some disadvantages. For example, RBAC is a time-consuming task, and it causes excessive administrator’s load due to the unique assignment of roles and permission to the users. On the other side, the management of ABAC is complex, and it is less secure than RBAC. The limitations and weaknesses of these traditional access control methods have been discussed in several papers [34,35,36,37]. It has been noted that these particular access control methods are not sufficient for a multi-layer IoT architecture [15]. To overcome the limitations and weaknesses of traditional access control methods, a trend suggests the need for hybrid models that merge existing access control models for the IoT. A hybrid model can use the advantages of individual methods. This paper discusses the issues and challenges of traditional access control mechanisms and provides theoretical guidance for required IoT access control mechanisms in a large-scale heterogeneous environment. Protocol-based and hybrid access control is the main focus of this paper. That said, we bring the critical issues of protocol and hybrid-based approaches in IoT access control in a single literature. The deployment of a hybrid access control scheme helps to use the advantages of different methods to authorize access activities in each layer of an IoT architecture. In addition, protocol-based is another method proposed for access control in IoT networks to address the heterogeneous network management issues. The focus of protocol and hybrid access control becomes further meaningful when contemplating a large-scale IoT system, for instance, the IIoT [38] settings. This paper also tries to show the convergence of IoT access control mechanisms for building secure IIoT infrastructure and the importance of protocol and hybrids access controls in such large-scale systems toward the emergence of a hierarchical access control building for them. The major contributions of this paper can be summarized as follows:

- We review the potential for employing protocol-based and hybrid access control for the IoT systems and how that can overcome the challenges of traditional access control mechanisms.
- Our work is intended to help understand how to converge such approaches to improve IoT access control efficiently. We focus on the key benefits and limitations of this integration.
- We provide an overview of the challenges and opportunities for building hierarchical access control for large-scale IoT systems (e.g., industrial IoT (IIoT) settings) with protocol and hybrid access control approaches. We also list the associated difficulties that should be addressed to make such an approach efficient in the future.

### 1.3. Methodology

In this paper, we provide a systematic analysis of the literature. We selected papers from a broader period of time. We include the papers that are relevant to the IoT and access control in general, but more thoroughly examine the papers that are relevant and close to IoT access control. We also examine papers that are relevant to the IIoT systems. Some other related papers that closely correlate to the primary motivation of the paper are also included. A range of venues is considered, including books chapters, journals, conferences and workshops, and articles from multiple disciplinary repositories (e.g., technical papers, reports, and arXiv documents).

In their abstract, we mostly search the keywords access control, authentication, authorization, Internet of Things (IoT), industrial Internet of Things (IIoT), access rights transfer, security, etc. Then, we evaluate the articles by examining whether the article illustrates an architecture, presents a survey, explores different access control mechanisms, etc. Of the 285 papers we reviewed, we found 130 papers are closely related to our study. Finally, we examine and inquire each paper against the fundamental purpose of the paper (i.e., protocol-based and hybrid access control for the IoT). For our case, Thompson Routers, computing classification system (ACM), and Google Scholar, are used.

### 1.4. Organization and Roadmap

The rest of the paper is organized as follows. First, in Section 2, we discuss the various approaches of ProBAC and HyBAC for the IoT systems. Then, in Section 3, we provide a discussion of the lesson learned and issues related to building a large-scale IoT system (e.g., IIoT) with the need for hierarchical access control based on ProBAC and HyBAC. Finally, we conclude the paper in Section 4.

## 2. Protocol-Based and Hybrid Access Control in the IoT

Several proposals discuss the various IoT architecture based on layers. For instance, a three-layer architecture is presented in [39]. The layers are perception, network, and application. However, to focus on more distinct aspects of the proposals, [40,41] present four- and five-layer IoT architectures. For the former (i.e., four-layer architecture), the layers are sensing, network, service, and application interface. For the latter (i.e., five-layer architecture), the layers are objects, object abstraction, service management, application, and business management. In addition, a few other approaches discuss IoT architecture based on cloud and fog computing systems [42,43,44,45]. On the one hand, a cloud-centric architecture delivers more flexibility and scalability for infrastructure, platform, and storage. On the other hand, a fog computing-based approach provides more flexibility to the data processing and analytics at the edge devices [46]. However, the choice of architecture depends upon the system’s requirements and the designer’s choice. In general, in this section, we consider a four-layer architecture when discussing various approaches for ProBAC and HyBAC models for IoT systems.

### 2.1. Protocol-Based Access Control (ProBAC)

Several approaches examine the need for access control mechanisms for the IoT, and a few of them discuss access control based on protocols. In Table 2, we provide a summary of the access control mechanisms for the IoT based on ProBAC.

For instance, Yan [47] discusses the potential research on data security for the IoT and explores the importance of access control to this context. A smart security protocol named intelligent service security application protocol (ISSAP) is introduced in this paper. It helps to reduce the overhead of data resources during communication and uses a data packet encapsulation mechanism. This mechanism combines cross-platform communications with encryption, signature, and authentication algorithms to provide IoT data security. However, the application of the proposed protocol in a real-world IoT scenario is not discussed, nor is the implementation provided.

Kothmayr et al. [48] present an approach for securing access control in IoT based on the DTLS protocol and existing internet standards. DTLS is based on the widespread TLS (transport layer security) protocol used to secure HTTPS for unreliable datagram transport. The proposed scheme is a standard two-way authentication-based secure architecture based on the RSA cryptosystem (a widely used public-key cryptography algorithm) that focuses on application-layer end-to-end security. The proposed protocol is situated between the transport and application layers. The authentication is done during the DTLS handshake (exchange of X.509 certificates) and the exchange of 2048-bit RSA keys. An extensive evaluation is performed to show the effectiveness of the proposed approach to message integrity, confidentiality, and authenticity with respect to three metrics, namely, energy usage, end-to-end latency, and memory overhead. The proposed scheme is designed to work over standard communication stacks over UDP/6LoWPAN. For IoT systems, it provides a lightweight solution for authentication and authorization.

Similar to [48], Sitenkov [49] presents a detailed discussion on IoT access control for the IETF (internet engineering task force) standard draft CoAP [50] based on DTLS for transport security. The CoAP is developed by the IETF Constrained RESTful Environments (CoRE) group based on the REST (and therefore on HTTP) message transfer protocol. A centralized approach is taken to store the corresponding access control information in the framework for the users for specific resources. Unlike [48], the author argues that the public key cryptography operation is computationally expensive for resource-constrained IoT devices, and therefore a lightweight symmetric key cryptography is used. In this framework, three types of agents are deployed. First, the constrained devices are noted as objects and called a resource server (RS) that hosts CoAP resources. Second, the subject is named the client (C), which connects to RS in order to access resources. Finally, a centralized server acts as a trusted anchor (TA), which stores the trust relations with various RS and at the same time holds an access control policy that regulates C’s access to the RS. During communication, in the beginning, C asks for a key to the TA; after obtaining the key, a DTLS handshake is performed between the C and the RS. The proposed framework is discussed, implemented and evaluated. Moreover, the framework’s efficiency over the DoS (denial of service) attack (e.g., a drain battery attack) is presented with evaluation efficiencies.

**Table 2:** Summary of Access Control Mechanisms for the IoT based on ProBAC.

Ref.	Purposes	Key Contribution	Implementation
[47]	Examining the employment of data security and access control for an IoT-based system.	Proposes a protocol called *Intelligent Service Security Application Protocol (ISSAP)* that uses a data packet encapsulation mechanism for IoT access control.	No
[48]	Building an access control model supported by DTLS.	Proposes an approach for securing IoT access control using DTLS protocol and existing Internet standards.	Yes
[49]	Employment of light-weight key management mechanism for securing IoT access control.	Proposes a centralized access control model using CoAP supported by DTLS for transport security.	Yes
[51]	Employment of lightweight key management mechanisms by avoiding resource expensive public key cryptography.	Proposes a flexible and delegation based authentication and authorization framework for constrained IoT devices.	Yes
[52]	Providing a holistic framework for securing SOA-based low power networks that are composed of constrained IoT devices.	Develops an access control framework considering the resource limited nature of the IoT devices using CoAP and Kerberos.	Yes
[53]	Building a smart gateway-based authentication and authorization method to prevent unauthorized access of medical information in an IoT-enabled smart healthcare facility.	Develops an access control framework combined with DTLS and CoAP-based authentication scheme for the IoT to provide high-end security in the datagram transport.	Yes
[54]	Examining an access control delegation using lightweight key management protocol.	Proposes a framework for delegating client authentication and authorization in a constrained environment using symmetric key cryptography.	No
[55]	Examining the use of PKI for IoT access control.	Develops an authorization and access control framework for IoT environment using a PKI scheme.	Yes
[56]	Examining the authentication in the life-cycle of an IoT device to secure access control.	Develops an *Authentication of Things (AoT)* protocol that addresses authentication and access control during the entire life-cycle of an IoT device.	Yes
[57]	Building an access control framework for resource-rich devices to perform expensive computation and processing tasks.	Proposes a cryptographic scheme for access control in IoT devices named *Efficient and Tiny Authentication (ETA)*.	No
[58]	Overcoming the overhead of heavy-weight PKI based cryptosystems within the resource limited IoT devices.	Proposes an end-to-end authentication framework for IoT by employing IBC and ECC.	No
[59]	Examining how to reduce the computational load requirements for sensor networks.	Proposes a user authentication protocol for WSNs using ECC and smart cards.	No
[60]	Examining how to reduce the computational load requirements for IoT systems.	Proposes a flexible and light-weight ECC based authentication scheme for resource constrained IoT systems.	No
[61]	Investigating the use of OAuth2 to build a federated and user-directed access control framework for the IoT.	Develops an access control framework for IoT based on OAuth.	Yes
[62]	Investigating the use OAuth2 to build an IoT access control framework.	Develops an access control framework, called *‘OAuth-IoT’*, for the IoT based on open standards OAuth protocol.	Yes
[63]	Building a unified access control scheme that integrates heterogeneous IoT devices and internet-based services.	Develops an IoT access control framework by integrating IoT devices with web-based services.	Yes
[64]	Designing a light-weight access control mechanism for IoT systems.	Discusses an access control enforcement mechanism within MQTT-based IoT systems.	Yes
[65]	Building an access control framework by providing fine-grained (remote) customization of access policies.	Proposes an architecture called *‘IoT-OAS’* which is an OAuth-Based authorization service architecture for secure services in IoT scenarios.	Yes
[66]	Examining light-weight access control frameworks to provide flexibility to existing Web-based services.	Proposes an access control framework for IoT based on CoAP.	No

**Figure 2:**
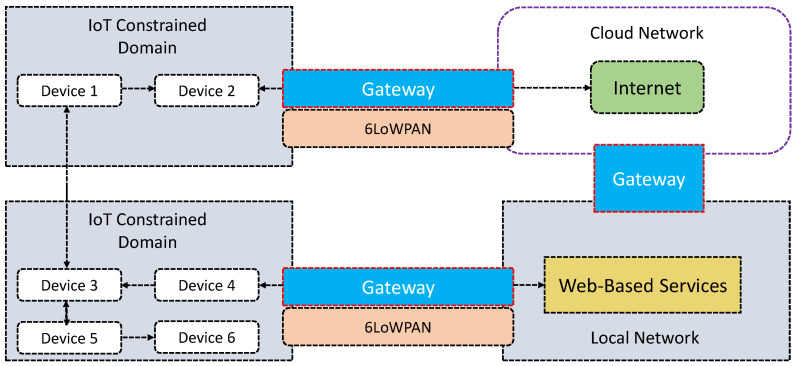
An outline of the proposed ProBAC model discussed in [51].

Hummen et al. [51] present a delegation-based authentication and authorization framework for IoT. Similar to [49], this proposal uses DTLS. The authors argue that the use of public key cryptography for peer authentication and key agreement purposes is not efficient, given the resource constrained nature in computation and battery capacity of the IoT devices. To alleviate these limitations, this framework uses a delegation architecture that offloads the expensive DTLS connection establishment to a centralized delegation server, which significantly reduces the resource requirements of DTLS-protected communication for constrained IoT devices. By doing so, the framework does not need to employ expensive public key cryptography for the connection establishment in the constrained devices and, therefore, only uses symmetric key cryptography for the protection of application data. Note, this framework separates the DTLS connection establishment and offloads the connection establishment to a delegation server. In Figure 2, we depict the simple overview of the proposal discussed in [51]. IoT devices are located in the ‘IoT Constrained Domain’. Devices in each domain can communicate with one another within the same or other domains for information sharing (delegation of access rights). In addition, web-based services can be accessed using a gateway (typically using 6LowPAN). Note, the entities that reside in the IoT constrained domain are equipped with the 6LoWPAN layer. A detailed experimental test-bed is presented with performance evaluation. For heterogeneous IoT environments, particularly for cross-domain authentication and authorization, these proposals (i.e., [49,51]) have significant value.

Pereira et al. [52] discuss a CoAP-based access control system focusing on low overhead for the IoT devices. Unlike [49], this proposal argues that the DTLS cannot accommodate a compliant and fine-grained mechanism for IoT access control, as it uses a high number of diverse key-pairs, which is difficult to process in IoT devices. At the same time, the management of key exchange mechanisms and administering a fine-grained access control is complex. The proposed solution is composed of Kerberos [67] and RADIUS (remote authentication dial-in user service) [68] and merges these two with the CoAP protocol to achieve a reliable access control framework for the IoT. The major motivation of this study is to provide a holistic framework for secure SOA (service-oriented architecture) based low power networks that are composed of resource constrained IoT devices. Furthermore, this framework separates the access control mechanisms from the communication security, which reduces the number of key pairs for DTLS encryption. Kerberos gives a lightweight protocol using symmetric-key cryptography, and RADIUS is a networking protocol employed for network authentication in wireless fields supporting access control, authentication, and accounting (AAA).

Kumar and Gandhi [53] present an IoT access control framework combined with DTLS and CoAP-based authentication design. The motivation of this study is to build a secure system (with strong authentication and authorization), using an intelligent gateway-based method. A use-case example of an IoT-enabled smart healthcare infrastructure is used in this paper. An enhanced DTLS is first used to perform authentication and authorization between the client and the gateway in this framework. Once the authentication and authorization processes are completed, a session update is used to connect the specific gateway and the server. Then, the client and the server can communicate over the smart gateway. The Cipher Block Chaining-Message Authentication Code (AES-CCM) is used to provide both authentication and confidentiality during the data transfer. The selection of a gateway is performed by mutual authentication using an ECDSA.

With a similar view of [51], Gerdes et al. [54] discuss a delegated authentication and authorization protocol for clients in IoT systems. The proposed protocol uses a lightweight solution and employs symmetric key cryptography for establishing a secure communication channel between the devices. It is, in particular, useful for the cross-domain environment. Unlike [51], in this approach, an IoT device is further able to delegate its access rights to other devices when sharing information through mutual authentication.

Pranata et al. [55] and Ning [69] present an authorization framework for an IoT environment, using a public key infrastructure (PKI) scheme. The motivation is to overcome what is lacking in the traditional internet network security, using minimal computing resources. Proposal [55] discusses a framework for authentication, authorization, and access control for IoT systems that use capability tokens with the advancement of PKI and encryption technologies focusing on the resource constrained environment, while at the same time aiming to use minimal computing resources. The proposed scheme also manages the identification and authorization permissions in the IoT environment, including consumer and service provider objects and authorization permissions management. Here, the capability token is used to assign access permissions for each user. The necessary access right permissions for each user accessing resources are written in XML [70]. Proposal [69] provides a similar view of [55] of using PKI between users and devices when performing access control operations.

Neto et al. [56] discuss an authorization framework for the IoT device based on their life cycle (i.e., from the production to the withdrawal of the device). They present an authentication of things (AoT), a suite of cryptographic protocols consolidated to address authentication throughout the complete life cycle of an IoT device. In this approach, identity-based cryptography (IBC) and attribute-based cryptography (ABC) are employed with the traditional ABAC mechanism. The use of these two cryptosystems is made, due to the nature of a certificate-free document, which does not provide any certificate-related overheads on the resource-constrained IoT devices. Significantly, the AoT allows mutual authentication in a cross-domain platform. The proposed framework is evaluated both analytically and experimentally. Similar to [56], Yavuz [57] presents a cryptographic scheme for IoT devices, named efficient and tiny authentication (ETA). However, unlike [56], this scheme does not consider the complete device life cycle and is dependent upon resource-rich devices (e.g., centralized server) to perform expensive computation and processing operations.

Markmann et al. [58] presents an end-to-end authentication framework for IoT by employing IBC and ECC. In this proposal, the authors argue that using IBC and ECC, the proposed framework has the advantage of overcoming the overhead of heavy-weight PKI based cryptosystem within the resource-constrained IoT devices. Unlike [56], this framework considers the federation of IoT sub-networks, where the sub-networks are connected to a dedicated gateway. No evaluation is given to support the framework.

With the similar view of [58], to address the key security issues and at the same time reduce computational load requirements for IoT devices, Yeh et al. [59] propose a user authentication protocol for WSNs using ECC and smart cards. Druml et al. [60] discuss the use of a flexible and lightweight ECC-based authentication scheme for resource-constrained IoT systems. This scheme enhances the concept of [59] by shifting parts of the computational intensity in ECC calculations from the resource-constrained IoT devices (which is represented by a smart card) to the authentication terminal hosted in resourceful computers. However, these approaches (i.e., [59,60]) provide little emphasis on the context of an IoT system and do not discuss an actual architecture.

Fremantle et al. [61] present an IoT access control framework for IoT supported by the Open Authorization (OAuth) [71] that is an alternative to using the MQTT [72] protocol. This proposal investigates the integration of OAuth2 within the MQTT protocol flow that supports federated and user-directed access control decisions for constrained environments. The proposed framework comprises four major components: MQTT broker, authorization server, web authorization tool, and devices. MQTT broker is based on a single Mosquitto broker, and it also contains a custom extension created to enable OAuth-based authentication and authorization. An authorization server is composed of the open-source WSO2 identity server. The web authorization tool helps a subject to create access tokens to authorize access to resources. In this framework, the edge IoT devices are built, using Arduino, and publish data to the MQTT broker. This approach of authentication and authorization is useful for lightweight and secure communication between IoT devices.

**Figure 3:**
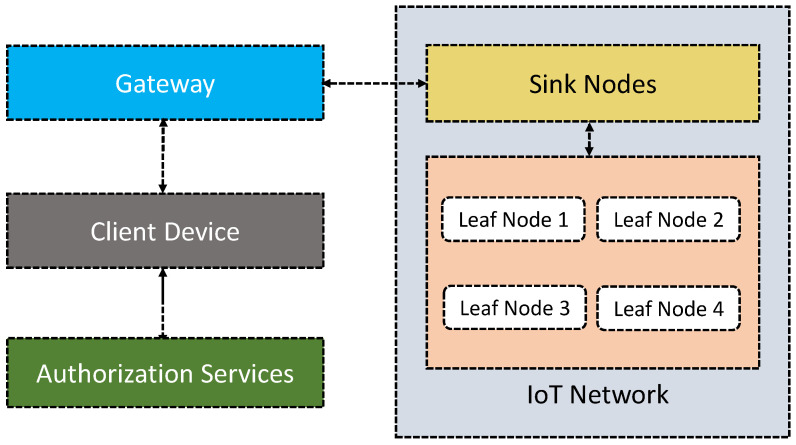
An outline of the proposed ProBAC model discussed in [62].

Similar to [61], Sciancalepore et al. [62] discuss an IoT access control framework called ‘OAuth-IoT’, based on open standards OAuth protocol (cf. Figure 3). It is intended to give secure authorization for HTTPS. The significant enrichment of the protocol is that it considers resource-constrained IoT devices, where high computational and bandwidth capabilities cannot be expected. It integrates existing open standards (e.g., OAuth 2.0) and the device’s resource-contained nature (e.g., limited capacity for processing, battery, and computation) for the IoT access control. Unlike [61], this proposal tries to make seamless interoperability between OAuth 2.0 and the IETF protocol stack that is lacking in present IoT access control solutions. The proposed framework is composed of four major components: the IoT network, gateway, client, and authorization server. The IoT network integrates many IoT devices, and the collected data from these devices are delivered to a ‘sink node’ (also known as a network coordinator), using low-power and short-range wireless communication technologies. The sink node is connected to the gateways and acts as a resourceful server. Clients request services from the IoT devices and access remote resources through OAuth 2.0 primitives. Finally, the authorization server controls and manages authorization mechanisms based on the OAuth 2.0 authorization framework.

**Figure 4:**
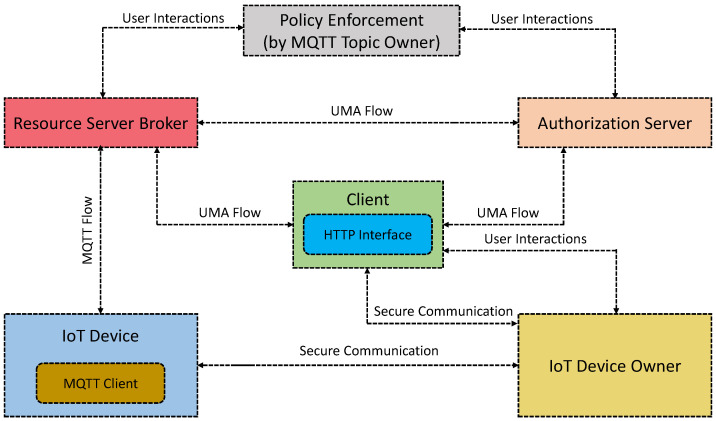
Information sharing with the different components of the ProBAC model presented in [63].

With a comparable design of [61], Cruz-Piris et al. [63] discuss an IoT access control approach by integrating IoT devices with web-based services by modeling certain IoT communication elements as resources (cf. Figure 4). In other words, the motivation is to build a unified access control scheme that would integrate heterogeneous IoT devices and internet-based services. MQTT is used for communication protocol and access control schemes, user-managed access (UMA) [73] (an existing OAuth 2.0 profile for internet services that offer a high level of granularity) is used. Colombo and Ferrari [64] present an IoT access control framework based on MQTT. Unlike [61], this approach is given with the ABAC that regulates message passing with corresponding access control methods based on the user’s preferences. The use of ABAC in the design provides much flexibility and becomes suitable for a broader range of application scenarios supported by attributes. Finally, a prototype is implemented with an initial evaluation.

Cirani et al. [65] present an architecture called ‘IoT-OAS’, which is an OAuth-based service authorization for IoT scenarios. The proposed architecture targets HTTP/CoAP services provided in an authorization framework with the combination of OAuth-based authorization service (OAS). In this architecture, access control is implemented inside the smart IoT devices. It explores the issue of scalability as an access control decision does not depend upon central entity leverage to make access control decisions within the devices. A performance evaluation is provided with simulation-based studies with Cooja (Contiki network simulator) [74]. Similar to [65], Wu et al. [66] present an access control framework for IoT based on CoAP and discusses its integration with HTTP to provide more flexibility to existing web-based services.

### 2.2. Hybrid Access Control (HyBAC)

In this section, we discuss the various HyBAC models for IoT systems. In Table 3, we provide a summary of the discussed HyBAC mechanisms.

**Figure 5:**
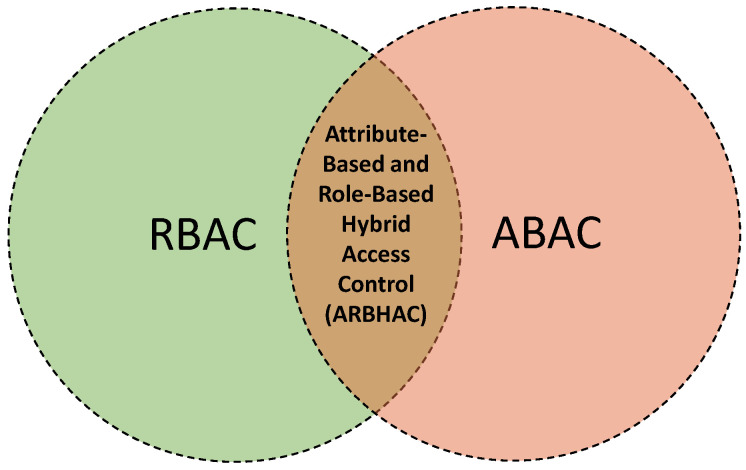
A conceptual representation of the proposed HyBAC model of [75].

For instance, Sun and Yin [75] present an *attribute-based and role-based hybrid access control (ARBHAC)* model for the IoT. A conceptual representation of the idea is illustrated in Figure 5. The model takes advantage of ABAC to satisfy large-scale dynamic users by specifying them into certain groups based on the roles. It also simplifies the complexity in permissions authorization and policy administration. After the attribute evaluations, each entity is given a specific role, and then RBAC is used to map the roles to permissions for accessing resources. Notably, this model suffers from well-known limitations, e.g., scale and complexity of both ABAC and RABC to an IoT context. No implementation of the model is provided.

**Figure 6:**
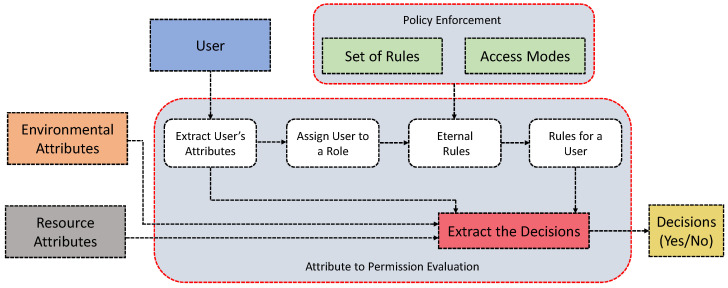
An HyBAC model for resource sharing in IoT discussed in [76].

In [76], Attia et al. discuss a hybrid access control model for highly dynamic IoT systems. This model combines RBAC and ABAC models to address scalability and flexibility issues to a fine-grained level. To benefit from the RBAC, this model defines roles, and every role has its specific permissions. The permissions are defined according to their access actions. To benefit from the ABAC, the identification of permissions considers the various attributes of subjects (e.g., users), objects (e.g., resources), and environments. In this way, the proposed model intends to scale the number of *things* and the dynamic contexts of the IoT systems. In Figure 6, we illustrate the outline of its working principle. For IoT, this framework can provide strong security based on RBAC features and, at the same time, a certain level of scalability, if possible, using the ABAC features of attributes.

Similar to the concept of [76] that combines RBAC and ABAC, in [77], the authors present a *policy RC-ABAC* (role-centric ABAC) model to address the need for efficient and flexible access control in internet-based resources. The proposed model is evaluated based on the four metrics: auditing, policy design, implementation, and maintenance. However, how these metrics are evaluated based on the proposed model is not clear. Therefore, only general discussions about these metrics are given without a broader discussion of the IoT context. Further, no implementation is provided.

Similar to the concept of [75,76], Aftab et al. [78] present a hybrid model for IoT-based smart applications that combines the features of traditional RBAC and ABAC models. The proposed scheme is called *hybrid access control (HAC)* that focuses on the secure localization of IoT-enabled smart vehicles. The motivation of this study is to develop an access control architecture for autonomous or driverless vehicles supported by an IoT infrastructure. A dynamic conflict of interest (COI) is used to reduce overload and latency in access control. The dynamic COI is combined with the proposed HAC model. The traditional RBAC model is enhanced by including the notion of ‘attributes’ within the RBAC features. The model takes advantage of high security supported by RBAC and dynamic features (using attributes) of ABAC. However, the model does not support the role hierarchy concept to control data access. No implementation details are presented.

**Figure 7:**
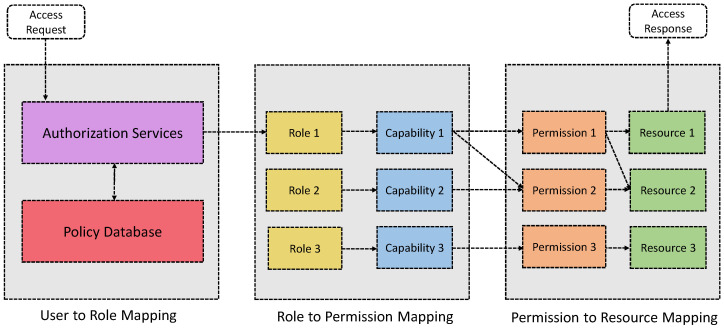
The proposed HyBAC model and information flow among entities presented in [79].

Pal et al. [79] present an HyBAC approach by combining the attributes, roles, and capabilities for resource-constrained IoT devices (cf. Figure 7). In this proposal, the IoT devices can make authorization decisions by themselves without relying upon a centralized authority. When a user requests a resource, they must supply some attributes to prove their identities. The identities are represented by a set of attributes and not by the unique concrete identities of the entities. Attributes can be the location, time, date, etc. Attributes are employed for role membership assignment and permission evaluation. In other words, it uses attributes to assign specific roles. The association of roles grants capabilities (i.e., access token) for specific access to a resource. Further, the issued capabilities may be parameterized depending upon the entity’s further attributes (and policies). Once satisfied, access is granted for authorized entities to access the resources. In an IoT context, it is significant, as it improves the edge intelligence of the IoT devices by locally evaluating authorization decisions at scale. The proposed HyBAC model is XACML driven. Finally, a detailed implementation of the model is provided.

Elhoseny et al. [80] present a hybrid model that combines cloud and IoT systems. The model is proposed to develop and optimize virtual machine selection in cloud-based IoT services for efficiently managing the high volume of data. The major purpose of this model is to enhance the performance of the IoT-enabled smart healthcare systems by reducing the computational execution time.

There have been some efforts made in combining a trust model with the ABAC model. For instance, Ouechtati and Azzouna [81] discuss a *trust-ABAC* model for IoT. This proposal considers the nature of IoT devices (especially the resource limitation issue) and reviews the critical aspects of an IoT system when proposing its access control model. Similar to [84], this model also stresses the need for a lightweight and secure access control mechanism in an IoT context. Fundamentally, the traditional ABAC and a trust model are combined. The authorization decision is highly dependent on the attributes of the different entities involved in making an access control decision. When an access request arrives for evaluation, the system decides the request based on access control policies defined for certain resources. The final access control decision is checked based on the provided attributes and the requester’s confidence value (i.e., the trust). Once both the conditions of the attributes and the corresponding trust value suffice, then access is given to the corresponding requester. The attribute condition is expressed as a set: {type,attribute,logicaloperator,value}, where type (e.g., int, bool, and string) is the type of the attribute (e.g., service, time, and place); logicaloperator is a set {≤,⩽,⩾,≥,etc.}, and the value is a certain value for a certain attribute, for instance, time = 7:00 a.m., place = university, etc. The access control logic is written in standard XACML.

With a similar approach of [81] (that combines trust and ABAC), Wang et al. [82] present an IoT access control model based on trust and attributes. However, unlike [81], which only considers the dynamic trust value, this model integrates both the static and dynamic trust attributes when making an authorization decision. This proposal aims to provide distributed security and control for fine-grained access control in IoT, using a trust attribute based on a node’s trust evaluation. Access requests are satisfied based on the supplied user’s attributes and the cumulative trust value. The proposed model comprises three major parts: the authentication module, trust evaluation module, and access decision module. They are responsible for user authentication, trust value calculation, and authentication and trust value to provide access to specific resources. This approach leads to the need for dynamic trust management in an IoT context supported by the attributes. Furthermore, in a multi-domain and heterogeneous IoT environment, this approach helps grant access permissions based on certain trust thresholds that must be satisfied on specific attributes.

Unlike [81,82], which combine a trust model with ABAC, Ray et al. [83] present an access control model combining a trust model and RBAC. The proposed access control model is designed for mobile cloud systems. It takes into consideration the dynamic context of the environment, where users move very fast from one application domain to another. This model formalizes the concept of trustworthy delegation for providing fine-grained access control in the highly dynamic context. Importantly, this model considers the aspects where the users dynamically acquire permissions from various application domains based on the required services. This model is an extension of [85], where trust levels are assigned to roles and which are then further assigned to permissions as in RABC. However, in [83], the specific problem of delegation is addressed based on the extension of the RBAC model and the trust-based access control model of [85]. This addresses the issues of dynamicity and inconstancy in controlling an access control delegation.

## 3. Discussion and Future Research Opportunities

Access control plays a vital role in securing resources from unauthorized access [86]. However, the need for access control in IoT is an emerging research area that needs more investigation to provide secure, scalable, lightweight, flexible, and trustworthy solutions [87,88,89]. Further, the flexibility in operation in different networks and operating systems are required for further improvement. For example, an access control solution that combines RBAC and ABAC properties can enhance the flexibility in security for a particular IoT system. To achieve this, in this paper, we examine the need for protocol-based (ProBAC) and hybrid (HyBAC) access control approaches for the IoT. We argue that ProBAC and HyBAC can provide more flexible and fine-grained access control overcoming the limitations of traditional access control mechanisms. For the former (i.e., ProBAC), integrating an IoT system for web-based services and modeling specific communication elements is significant. For the latter (i.e., HyBAC), two or more access control proposals to provide a better solution can be re-examined. The above section studies the recent approaches to analyze relevant proposals focusing on ProBAC and HyBAC.

We note that, at present, no complete and coherent access control solution covers every aspect of an IoT architecture by specifying its requirements [90,91]. Several proposals discuss the need for IoT access control and security-specific requirements, based on dedicated frameworks and particular use case examples. Access control in IoT is application specific, and it requires reliable infrastructures and given contexts within which it will function. This is also limited to specific access control proposals for constrained IoT devices (e.g., battery, memory, and processing capability) and their service-specific applications [10,92,93]. Recently, there has been a trend that focuses on the need for combining two or more access control proposals for IoT access control. It can come from the protocol level or integrating various access control mechanisms into a single mechanism. For instance, trust is integrated into ABAC to improve system functionality, scalability, security when accessing a resource [94]. In this case, the concept of trust is considered an attribute to enforce access control rules and policy specifications. In [95], the dynamic nature of attributes is analyzed to provide flexibility in access control on fog-based IoT networks. The concept of fog computing is integrated with ABAC that helps to make authorization decisions examining the user’s mobility. In [96], ABAC properties are further enhanced to the internet of multimedia things (IoMT) to provide multimedia data security and privacy.

**Figure 8:**
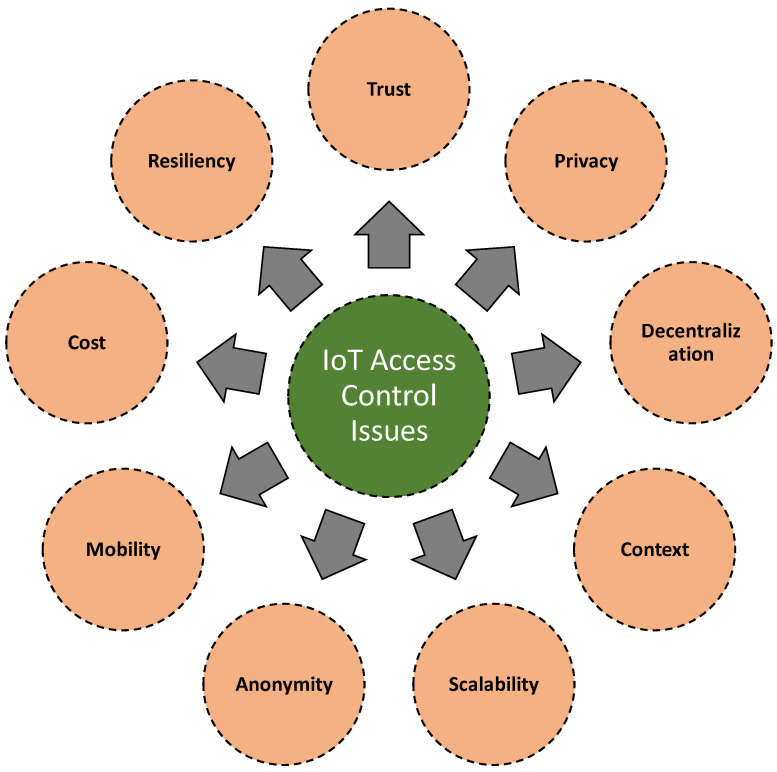
A list of significant issues associated with IoT access control.

Security solutions can be enforced at different levels in an IoT architecture. In other words, it is significant to see a hierarchical access control solution for sensitive information at different levels based on their security needs. Here, the term hierarchical denotes the association of entities in a hierarchy [97,98]. We also note that based on the device’s constraint, resource limitations, dynamic characteristics, and various other challenges (e.g., multiple jurisdictions and heterogeneity in services and applications) present in an IoT system, the need for access control is different in various layers of an IoT architecture [99,100]. For instance, the access control needs in the network layer should be different from the access control needs in an application layer. That said, in an application layer, access control is more restricted to authorized users, but in a network layer, access control must satisfy the prerequisites of secure communication. This further signifies the need for access control based on protocols and their adaptability in different layers in an IoT architecture [14]. In Figure 8, we illustrate some identified open issues associated with the IoT access control.

We have noted that there is a significant potential to employ ProBAC and HyBAC models for large-scale adaptation of the IoT systems. ProBAC and HyBAC are noticeable to use, among others, to address the issues of scalability, resource-constrained nature of the environment, accessibility, concurrency, interoperability, and the heterogeneous specifications of networks and devices. Some of the ProBAC models offer a flexible and more scalable approach, but at the same time, they lack usability. HyBAC is flexible and adaptable in many cases for IoT systems, but combining two models sometimes requires addressing the significant overhead concerns in administering access control. The distributed approach using capability (i.e., access tokens) combined with the ABAC is more flexible for effective and efficient authorization at the edge IoT devices.

In recent years, we have observed that IoT applications are gaining much popularity in industrial sectors. For instance, industrial IoT (i.e., IIoT) discusses the communication in machine-to-machine (M2M) and associated industrial communication technologies for more flexible and adaptive automation applications [101]. This is to note that the terms IoT and IIoT cannot be used interchangeably. In general, IoT is considered a web for the machines that allows *things* (e.g., users, devices, applications, and services) to exchange information with one another using that platform. IIoT can be seen as the fundamentals of digital infrastructure connecting all the industrial assets (e.g., machines and control systems) with the information systems as well as the business processes at a large scale. It emphasizes that the communication in IIoT is machine-oriented [102,103]. Our study shows a clear vision for the potential research opportunity to integrate and interconnect different access control approaches (ProBAC and HyBAC) to support hierarchical access control for the IIoT sectors at scale.

**Figure 9:**
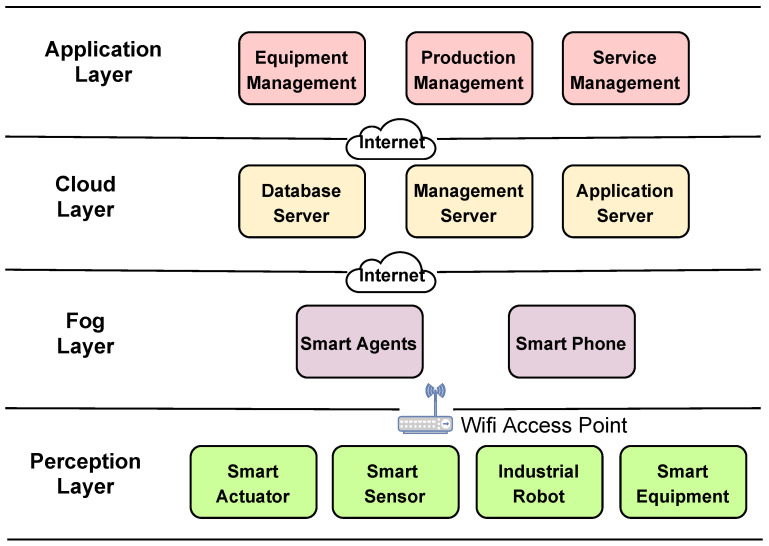
Outline of a four-layer IIoT architecture [111].

Moreover, Industry 4.0 (i.e., the fourth industrial revolution) focuses heavily on interconnectivity and automation, which enhanced the properties of an IIoT system at scale with many extensions and opportunities [104]. For example, Industry 4.0 enables intelligence production in the cloud and big data processing, interconnectivity, and automation among devices in manufacturing companies [105]. IIoT can be seen as a platform, where computing technologies can connect physical things to networks. Industry 4.0 provides smart manufacturing in which intelligent devices can collect data and share the data amongst intended peers (i.e., authorized). IIoT integration in Industry 4.0 shows that manufacturing devices that are wirelessly connected to the internet (or an internal network) can provide more flexibility in automation [106,107]. Therefore, in an industrial network, IIoT distinguishes manufacturing devices from consumer devices, describing M2M communication. This feature provides high-quality connectivity and messaging, and inter-operable interactions between machines, where access control has paramount significance [108,109]. Some benefits of Industry 4.0 and IIoT are complex task sharing, decision making based on collected data, and remote access to machinery. Due to the massive connectivity of devices in IIoT and the data collection/sharing capability, there is a need for hierarchical access control in industrial environments. Research has shown that traditional access control methods are not enough for IIoT networks [110], which is the same for the conventional IoT environments. While different architectures have been proposed for IoT, no generic architecture can be referenced as a standard model where an access control model can be employed uniquely. Among various layered architectures for the IIoT, for our purpose, we consider a four-layer architecture (discussed in [111]) to explain the hierarchical access control in IIoT networks. This architecture composes of perception, fog, cloud, and application layers. Each layer of this architecture needs a specific set of security countermeasures. That is, a generic access control model for each layer is not sufficient. This four-layer architecture, illustrated in Figure 9, is employed in our paper to discuss the requirements of access controls in each layer. We emphasize that the access control mechanisms should be applied to different layers of an IIoT architecture, and hence, a hierarchical access control model is required. Recall that ProBAC and HyBAC can be combined and employed based on the system’s requirements and context of the architecture in which it will function. In Figure 9, the typical activities of each layer are as follows:

- **The perception layer** collects information from industrial devices, such as sensors (e.g., temperature), smart equipment (e.g., robots), and smart actuators. This layer is responsible for data collection, command execution, and authentication of data and devices. The data collected in this layer are transferred to the fog layer, using an edge gateway (e.g., Wi-Fi access point).
- **The fog layer** comprises fog nodes, which are devices that are the first point of contact to IIoT end devices. Fog nodes can be intelligent devices, such as tablets, smartphones, etc. IIoT devices in the perception layer forward raw data to their nearest fog node.
- **The cloud layer** includes different servers, such as a database server and application server. The cloud stores a massive amount of data and provides big data analysis. It also helps to communicate over various networks domains that are necessary for an IIoT context.
- **The application layer** consists of users who work with intelligent terminals and manage the industrial workflow. Decision making provided in this layer is based on the output of big data analysis, and it aims to improve the quality of products and services in the industry.

Communication between devices within the same and across layers should be monitored to ensure that only trusted fog nodes can access IIoT devices in the perception layer. IIoT devices are resource-constrained, and access control and authentication need computational resources. Hence, these computationally intensive operations are outsourced to the fog nodes [111]. This emphasizes the lightweight access control solutions for the edge IIoT devices.

IIoT is responsible for collecting data from different sensors embedded in the production devices, and it helps in the automation of advanced manufacturing machines. However, there is high heterogeneity of IIoT standards, techniques, and protocols, and hence, access control in this environment is challenging [101,112,113]. Due to the complexity of the IIoT environment as discussed above, the employment of ProBAC and HyBAC is beneficial to improve the security of this emerging technology. For example, access control policies are necessary for IIoT applications to control users who access the applications and query data [114,115]. In addition, IIoT must deploy access control mechanisms on the cloud to control access to the data stored on the cloud. Encryption with access control can be used for cloud-based IIoT applications [116,117,118]. Different protocols support M2M communication in IIoT, and hence, protocol-based access control can be used in the hybrid method to improve the efficiency of the access control mechanism [119]. It is crucial when considering the operations over different jurisdictions combining heterogeneous networks.

These further demand trust between various entities in a highly dynamic and scalable system, such as the IoT [120,121]. Several proposals address the significance of trust and present specific access control scenarios based on a trust model. However, most of the approaches enhance the traditional distributed trust management systems for the IoT, lacking the proper need for trust in an IoT context and considering the dynamicity present in the system. To this end, in the future, the distributed ledger technology, e.g., blockchain, can provide a solution [122,123]. Furthermore, it reinforces the decentralized requirement of access control in both IoT and IIoT systems at scale [124]. For instance, hybrid access control is proposed in [125] for IIoT networks. In this paper, the authors utilized a blockchain-based access control under edge computing. The edge layer of their access control mechanism is based on the Bloom filter, designed for identity management. Further, a lightweight secret key agreement protocol based on a self-authenticated public key is employed for securing access control at the edge level. That is, the protocol is deployed for secure communication in edge devices, ensuring data authentication, auditability, and confidentiality. Another blockchain-based access control model is proposed in [126] that focuses on the 5G-enabled IIoT systems. 5G-enabled IIoT introduces more security and privacy challenges due to its high mobility and dynamicity. A consortium blockchain-based access control framework is used in [126]. Three chain-codes are utilized in this framework; namely, policy management chaincode (PMC), access control chaincode (ACC), and credit evaluation chaincode (CEC), to secure authentication and authorization.

In summary, it can be seen that there is a need for protecting IoT/IIoT systems from unauthorized users, services, and applications by enforcing appropriate access control mechanisms that satisfy the various characteristics and requirements of an IoT system [127,128]. It could not be fixed by using simple software patches or applying heavy-weight security mechanisms inside the resource-constrained IoT devices. It requires dedicated access control architecture, lightweight security mechanisms, secure communication protocols, and appropriate security protocols policy management. Moreover, the scope and variety of recent technological developments impose sophisticated constraints for authentication and authorization in IoT/IIoT systems that are not supported by earlier security frameworks [129,130]. We argue that ProBAC and HyBAC have excellent potential in this domain that can also minimize the overhead created by many security mechanisms for the IoT/IIoT. These mechanisms can be enforced in various ways in access control and policy management, e.g., in back-end management, secure design and development practices, or even at an application level.

## 4. Conclusions

An IoT system comprises various devices, and the devices may have distinct operating systems, hardware, and software configurations, as well as perform in heterogeneous communication networks. Typically, the IoT devices are resource-constrained in nature, i.e., short battery power, limited processing speed, and insufficient memory capacity. These limitations do not allow for the employment of traditional heavy-weight security architectures for the IoT. Therefore, security in IoT is a prime factor in providing better services and applications to the users considering all these limitations. Several surveys address IoT access control issues, but the specific case of protocol-based and hybrid access control is missing in the recent literature.

This paper presented an extensive and systematic review of the protocol and hybrid-based access control models for IoT systems. We showed the trend for more flexible and fine-grained access control by using various protocols and combining two or more access control models for IoT. Our study also focused on building large-scale IoT systems (e.g., IIoT designs) with the need for hierarchical access control with the protocol and hybrid-based approaches. Our study showed the flexibility of adopting such an approach to the IIoT systems at scale. That said, we provided insight into the need for hierarchical access control for large-scale IoT (e.g., IIoT) systems. We explained how this could be achieved, using the protocol and hybrid-based access control. Finally, we listed the open issues and future research directions of such integration.

## Figures and Tables

**Table 1:** Previous reviews on IoT access control and their comparison with our work.

References	ProBAC	HyBAC
[23]	✗	✗
[24]	✗	✗
[25]	✗	✗
[26]	✗	✗
[27]	✗	✗
[28]	✗	✗
[29]	✗	✗
[30]	✗	✗
[31]	✗	✗
[32]	✗	✗
[33]	✗	✗
[Our Work]		

**Table 3:** Summary of Access Control Mechanisms for the IoT based on HyBAC.

Ref.	Purposes	Key Contribution	Implementation
[75]	Combining RBAC and ABAC models for IoT access control.	Proposes an *Attribute-Based and Role-Based Hybrid Access Control (ARBHAC)* model for the large-scale dynamics users to improve policy management.	No
[76]	Combining RBAC and ABAC models to address the issues of scalability and flexibility in IoT access control to a fine-grained level.	Proposes an access control model for the IoT combining with properties of both RBAC and ABAC models.	No
[77]	Combining RBAC and ABAC models for IoT access control.	Presents a *Policy RC-ABAC* (Role-Centric ABAC) model to address the need for fine-grained and flexible access control for IoT systems.	No
[78]	Combining RBAC and ABAC models for access control in autonomous vehicles.	Proposes an access control architecture called *Hybrid Access Control (HAC)* that focuses on the secure localization of IoT-enabled smart vehicles.	No
[79]	Combining RBAC, ABAC, and CapBAC models for light-weight access control at edge IoT devices.	Attributes are employed for role membership assignment and access control permission evaluation. The membership of roles grants capabilities (i.e., access tokens) for specific access to a resource.	Yes
[80]	Building an access control model for cloud-based IoT services.	Proposes an access control architecture for IoT-enabled smart healthcare systems to handle a big amount of data without human intervention.	No
[81]	Combining trust and ABAC models for IoT access control.	Develops a *Trust-ABAC* model for fine-grained access control in IoT systems based on provided attributes and trust value of a service requesting entity.	Yes
[82]	Combining the notion of trust (i.e. trust value) and ABAC model for IoT access control.	Proposes a distributed, and flexible access control model for IoT using trust attributes that are based on an entity’s trust evaluation.	No
[83]	Combining trust and RBAC models for IoT access control.	Develops a model for mobile cloud-based IoT infrastructure to provide fine-grained access control for complex IoT systems that depends upon a highly dynamic context.	Yes

## Data Availability

Not applicable.
